# School-based diet and physical activity interventions and weight-related outcomes among children and adolescents in Africa: a systematic review and meta-analysis

**DOI:** 10.1186/s12889-026-29255-3

**Published:** 2026-08-29

**Authors:** Barbara Adimchinobi Onyeonu, Favour Chiamaka Kalu, Kosisochukwu Chidinma Igbokwe, Uzoamaka Victoria Thomspon, Favour Chidinma Okezie, Anuoluwapo Funmilayo Taiwo, Gideon Onyedikachi Iheme

**Affiliations:** 1 Department of Human Nutrition and Dietetics, University of Ibadan, Ibadan, Nigeria; 2 Department of Human Nutrition and Dietetics, Michael Okpara University of Agriculture, Umudike, Nigeria; 3 Department of Medicine and Surgery, Abia State Teaching Hospital, Aba, Nigeria; 4 Department of Food Studies, Nutrition and Dietetics, Uppsala University, Uppsala, SE-751 22 Sweden

**Keywords:** School aged children, Obesity, weight reduction, interventions, management/treatment, Africa

## Abstract

**Introduction:**

The increasing prevalence of Obesity and associated health risks in LMICs underscores the urgent need to adopt an integrated approach to Obesity prevention and management. This systematic review aims to assess the impact of school-based diet and physical activity interventions on weight-related outcomes among children and adolescents in Africa.

**Methods:**

This systematic review was registered with PROSPERO (CRD42023450307) and reported according to the PRISMA statement. A comprehensive literature search was conducted across three databases, PubMed, Google Scholar and African Journals Online from January 2000 to December 2025.

**Result:**

Our search identified a total of 11 eligible studies, with modest to low risk of bias, of which majority of the studies were published within the last decade (63.6%), and conducted in South Africa (72.7%). These studies were predominantly quasi-experimental (70%) or cluster randomized control trail (18.2%) in design. The majority (73%) interventions were delivered in primary school settings and participants were mainly engaged in group activities. The bulk of the interventions were multi-component, comprising physical activity, alongside nutrition, and other health/behavioural interventions. Overall, the studies reported modest improvements in elevated adiposity and maintenance of normal weight status in the intervention groups. However, this translated into a non-significant effect in the meta-analysis of nine intervention comparisons on BMI-for-age (MD = 0.03, 95% CI: −0.24 to 0.29; *p* = 0.813; I² = 69.0%), although combined nutrition and physical activity interventions showed reductions in a few comparisons.

**Conclusion:**

School-based diet and physical activity interventions show potential to improve adiposity and maintain healthy weight status among African children and adolescents, particularly when combined. However, the overall effect on BMI-for-age was not significant, highlighting the need for more geographically diverse and methodologically robust evidence across Africa.

**Supplementary Information:**

The online version contains supplementary material available at https://doi.org/10.1186/s12889-026-29255-3.

## Introduction

Between 1990 and 2022, the prevalence of Obesity has more than doubled [1]. A new global record was established when obesity exceeded underweight as the most prevalent form of malnutrition among school-aged children and adolescents [2]. Currently, 1 in 5 children, and 1 in 10 children and adolescents in the world are affected by overweight and obesity, respectively [2]. Although low-income regions like Sub-Saharan Africa and South Asia do not yet align with these global statistics [2], the prevalence of overweight and obesity is increasing at a higher rate than in several high-burden countries, having quadrupled in the last two decades [2, 3]. The substantial challenge of undernutrition among children in low- and middle-income countries (LMICs) is increasingly compounded by the double burden of malnutrition, underscoring the need to strengthen preventive strategies aimed at addressing the rising prevalence of overweight and obesity.

Integrating positive lifestyle behaviours during childhood has been associated with improved overall well-being, the establishment of sustained healthy habits, and a reduced risk of obesity and non-communicable diseases across the life course [4, 5]. Given that children spend up to 20% of their waking hours in classroom-related activities [6], and schools are recognised as institutions that provide a supportive learning environment where behaviours are actively formed and internalised, school-based interventions represent a strategic avenue for promoting positive lifestyle behaviours capable of fostering the maintenance of a healthy weight or gradual reversal of excess weight.

The global commitment to addressing the scourge of obesity is widely acknowledged. The World Health Organisation (WHO) Acceleration Plan to Stop Obesity represents a collective consensus and adoption by all United Nations member states on recommended structural approaches to tackling Obesity, including an intent to translate these recommendations into policies and implementation within local and national contexts [7]. Furthermore, global authorities have consistently identified improvements in nutrition and physical activity as the primary approach for obesity prevention in children and adolescents, with school touted as the most important setting for delivering interventions for this age group [8].

Although the gaps and challenges associated with policy development and implementation vary across geographical regions and income settings [9, 10], the evidence base for obesity interventions among children and adolescents in Africa is still emerging. While multiple high-quality reviews on obesity prevention have been conducted in other regions and contexts, the available evidence relevant to Africa either encompasses broader geographical regions or intervention settings [11, 12] or is now due for updating [11–13]. This study aims to synthesise evidence on the effectiveness of school-based dietary and physical activity interventions on weight outcomes among children and adolescents in Africa.

## Methods

This study, registered in PROSPERO (registration no CRD42023450307), was compiled following Preferred Reporting Items for Systematic Reviews and Meta-Analyses [PRISMA] guidelines [14].

### Search methods for the identification of studies

A comprehensive search of three databases, including PubMed, Google Scholar, and African Journals Online (AJOL), was conducted between 2000 and 2025 to identify publications within the study’s scope. The reference lists of the selected articles were screened to identify potentially relevant studies that could have been omitted during the electronic search. The English descriptors included the search terms in combination with the Boolean “OR” and “AND” operators; Overweight OR Obesity OR Weight-related outcomes OR Weight maintenance OR Weight gain OR Weight status OR Body mass index OR BMI OR Adiposity OR Fat mass AND School-based OR School intervention OR School program OR School health OR Diet OR Dietary intervention OR Nutrition OR Nutrition education OR Healthy eating OR Physical activity OR Exercise OR Physical education OR Lifestyle intervention AND Children OR Child OR Adolescents OR Adolescent OR Youth OR Students AND Africa. Details of the comprehensive search strategy is available in supplementary file 1.

### Study selection process and data extraction

Two reviewers independently screened all titles and abstracts for compliance with the eligibility criteria. The full text of the pre-selected articles was further evaluated at this stage. Any disagreements regarding eligibility were resolved through discussion and, if necessary, the intervention of a third reviewer. The inclusion criteria were defined based on the PICOS (Population, Intervention, Comparison, Outcome, and Study Design) framework.

#### Population

School aged children and adolescent in Africa

#### Intervention

Single-component or multicomponent lifestyle interventions comprising dietary interventions, physical activity interventions, combined dietary and physical activity interventions, or these delivered alongside other intervention components.

#### Comparison

Participants or groups who did not receive any intervention or were subjected to standard/basic care were compared to those who received some form of intervention. Alternatively, the baseline status of participants or groups prior to the intervention was compared to their post-intervention status, for studies with no comparator.

#### Outcome

The outcomes of interest were weight-related measures, with BMI-for-age and BMI as the primary outcomes, alongside other anthropometric indicators reported across the included studies.

#### Study design

Quantitative intervention studies (randomized controlled trials, quasi-experimental studies, controlled before and after studies, and other intervention studies) published between 2000 and 2025 that evaluated the above-stated interventions for improving weight-related outcomes.

Information extracted from the selected articles included: author’s name, study location, Sample size, study design/technique, study instruments, intervention related components and associated implementation strategies, as well as the outcome of the intervention.

### Study quality/risk-of-bias assessment

Risks of bias were assessed independently by two reviewers. Due to the heterogeneity of the study designs in the selected studies, eight questions were extracted from the Joanna Briggs Institute (JBI) critical appraisal tools for quasi-experimental studies [15] and randomized controlled trials [16] to evaluate the methodological quality of the studies. These eight questions were focused on evaluating: inclusion of a comparator group (1), similarity of comparison groups (2) and treatment/care received apart from the intervention (3), multiple measurement of outcomes before and after intervention/exposure (4), consistency (5) and reliability (6) in the form and nature of measurement across all comparison groups, follow-up completion or adequate description of differences (7) and appropriateness of employed statistical analysis (8). The response options to each question are ‘yes’, ‘no’, ‘unclear’, and ‘NA’ (non-applicable). The criteria met for each question were assigned 1 point; only studies that did not garner at least 3 points out of a possible 8 points were regarded as of low quality and excluded from the survey. This low benchmark was to account for the disparities in study designs of the included studies.

### Variables and data analysis

Data extracted from the included studies were organized into four main categories. First, study characteristics, including study location and population, study design, sampling methods, and variables or study instruments, were summarized descriptively to provide an overview of the included studies.

Second, intervention characteristics were extracted and summarized, including the intervention type (nutrition alone, physical activity alone, combined nutrition and physical activity, and other multicomponent interventions), setting or mode of delivery, duration and frequency, and delivery personnel were reported.

Third, outcome data were extracted, focusing on the primary outcomes of interest, namely anthropometric and weight-related measures. These outcomes were reported as means and standard deviations where available, and changes between baseline and post-intervention measurements were calculated. For uncontrolled studies, comparisons were based on baseline and post-intervention values within the same participants, whereas for controlled studies, intervention effects were assessed by comparing changes in the intervention group with those observed in the comparator group. Outcome interpretation was guided by the baseline weight status of the study population. For studies involving participants with overweight or obesity, reductions in adiposity-related measures particularly BMI-for-age z-score or BMI, but also body weight, waist circumference, were considered indicative of improved outcomes (“favourable outcome”). For studies involving predominantly normal-weight populations, maintenance of healthy weight status and anthropometric measures within normal ranges, as well as the prevention of excess adiposity gain, were also considered as “favourable outcomes”. These reductions in adiposity, or maintenance of a healthy adiposity status, were considered “favourable or desirable” when the intervention group demonstrated greater improvement or better maintenance than the comparator group. Outcomes were classified as **“**Neutral” when both groups maintained a normal adiposity status without a meaningful between-group advantage, and **“**Unfavourable” when the intervention failed to reduce elevated adiposity measures or performed worse than the comparator group. These findings were interpreted in accordance to the direction and magnitude of the change in mean of the intervention group relative to the comparator group.

Finally, while all descriptive analyses were done using IBM SPSS Version 28 [17], the meta-analysis was conducted using R (Version 4.3.3) [18] using the meta package. For studies with intervention and comparator groups reporting continuous BMI-for-age z-score values and their corresponding standard deviation, their pooled mean differences (MDs) with 95% confidence intervals (CIs) were estimated using a random-effects model with the inverse-variance method, restricted maximum likelihood (REML) estimator, and Hartung–Knapp adjustment. Where multiple intervention groups exist for a particular study, all intervention groups were treated distinctly. Other case specific adjustments included the splitting of shared control groups for multiple interventions and combination of sex-specific results. Statistical heterogeneity was assessed using Cochran’s Q test, τ², and the I² statistic. Forest plots were generated to present individual and pooled effect estimates.

## Results

**Fig. 1:**
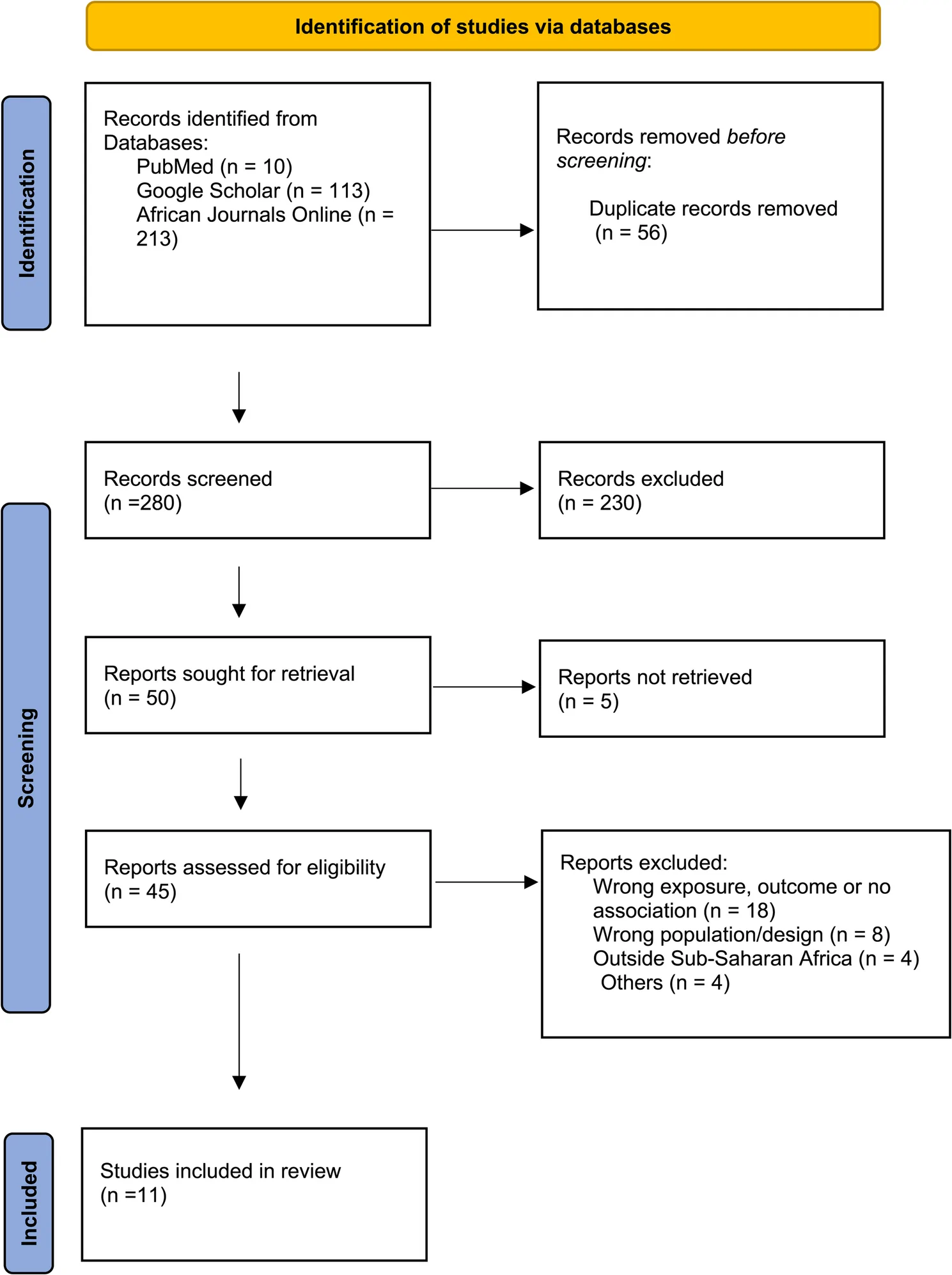
PRISMA flow chart of article selection

The database search yielded a total of 336 original articles, which was later reduced to 280 articles after the initial removal of duplicates found across the multiple databases. Screening of abstract and full texts resulted in the further removal of 269 articles which did not meet the outlined inclusion criteria. At the end of the study selection process, 11 articles in all were found eligible for inclusion in the review (Fig. 1).

### Description of selected studies

An overview of the included studies is presented in Table 1, and a descriptive summary of study characteristics is available in supplementary Table 2. The majority (63.6%) of the studies included in this review were published between 2013 and 2022, while 36.4% of them were published between 2002 and 2012. They were conducted in three countries, with the majority (72.7%) of the studies originating from South Africa, followed by Tunisia (18.2%), and the Democratic Republic of the Congo (9.1).

**Table 1:** Characteristics of included studies

Authors	Country/ Location	Study population	Sample size	Study Designs/ technique	Variables	Study instruments
Naidoo et al. [19]	South Africa	School aged children (SAC) and adolescents (10–15 years)	185	Quasi-experimental	Anthropometric assessments (weight, height, body mass index (BMI))	Self-reported NAP questionnaire, semi-structured interview; Eurofit physical fitness test battery
Draper et al. [20]	South Africa/ Johannesburg	School-age children (age not reported)	508	Quasi-experimental	Anthropometric assessment (weight, height)	Questionnaire; focus group discussion; validated physical activity questionnaire
Kemp and Pienaar [21]	South Africa/ Potchefstroom	School-age children (9–12 years)	38	Quasi-experimental	Anthropometric assessment and body composition; self-perception	Harter self-perception profile questionnaire (interview-based)
Monyeki et al. [22]	South Africa/ Gauteng	School-age children (9–13 years) Secondary school	322	Quasi-experimental	Anthropometric assessment (weight, height, BMI, % body fat)	Not stated
Regaieg et al. [23]	Tunisia/ Sfax	Adolescents (12–14 years)	28	Quasi-experimental	Anthropometric assessment (weight, height, BMI, WC); body composition fat free mass (FFM) and fat mass (FM)	Bioelectrical impedance equipment; Ergometer
Maatoug et al. [24]	Tunisia/ Sousse	7th and 8th grades School Children with mean age of 13.5	585	Quasi-experimental	Anthropometric assessment (weight, height, BMI), physical activity, dietary habits	Questionnaire, 24 h food recall, physical activity recall questionnaire
Muller et al. [25]	South Africa/ Port Elizabeth	Primary-school age children (9–14 years)	746	Cluster RCT	Anthropometric assessment; cardio-respiratory fitness	Self-reported physical activity questionnaire; 20 m shuttle run test
Grace et al. [26]	South Africa/ eThekwini Municipality	Adolescents in grade 8 and 9 (13–16 years)	41	Quasi-experimental	Anthropometric assessment (weight, height, BMI); blood pressure; fasting biochemical variables (glucose, HDL-C, LDL-C, TG, TC etc.)	Borg RPE scale (a scale report index that reflex perceived exercise intensity)
Wanghi et al. [27]	Democratic republic of Congo/ Kinshasa	Adolescents (12–17 years)	64	RCT	Anthropometric assesment (weight, height, BMI)	Not stated
Nqweniso et al. [28]	South Africa/ Gqeberha region	grade-4 School-age children (8-11years)	898	Cluster RCT	Anthropometric assessment (weight, height, BMI-for-age, % body fat)	Self report questionnaire (9 item)
Idamokoro et al. [29]	South Africa/ Raymond Mhlaba Municipality	School-age children (7–8 years)	93	Quasi-experimental	Anthropometric assessment	Not stated

The sample sizes ranged from 28 to 1,304 participants, with approximately 45.5% of the studies having between 101 and 1,000 participants, and 45.5% of the studies reporting fewer than 100 participants. The majority (90.9%) of the studies included both male and female participants, and 9.1% on males only.

Over 70% of the studies used a quasi-experimental study design, followed by a cluster randomized controlled trial (18.2) and randomized controlled trial (RCT) (9.1%).

### Quality assessment of included studies

**Fig. 2:**
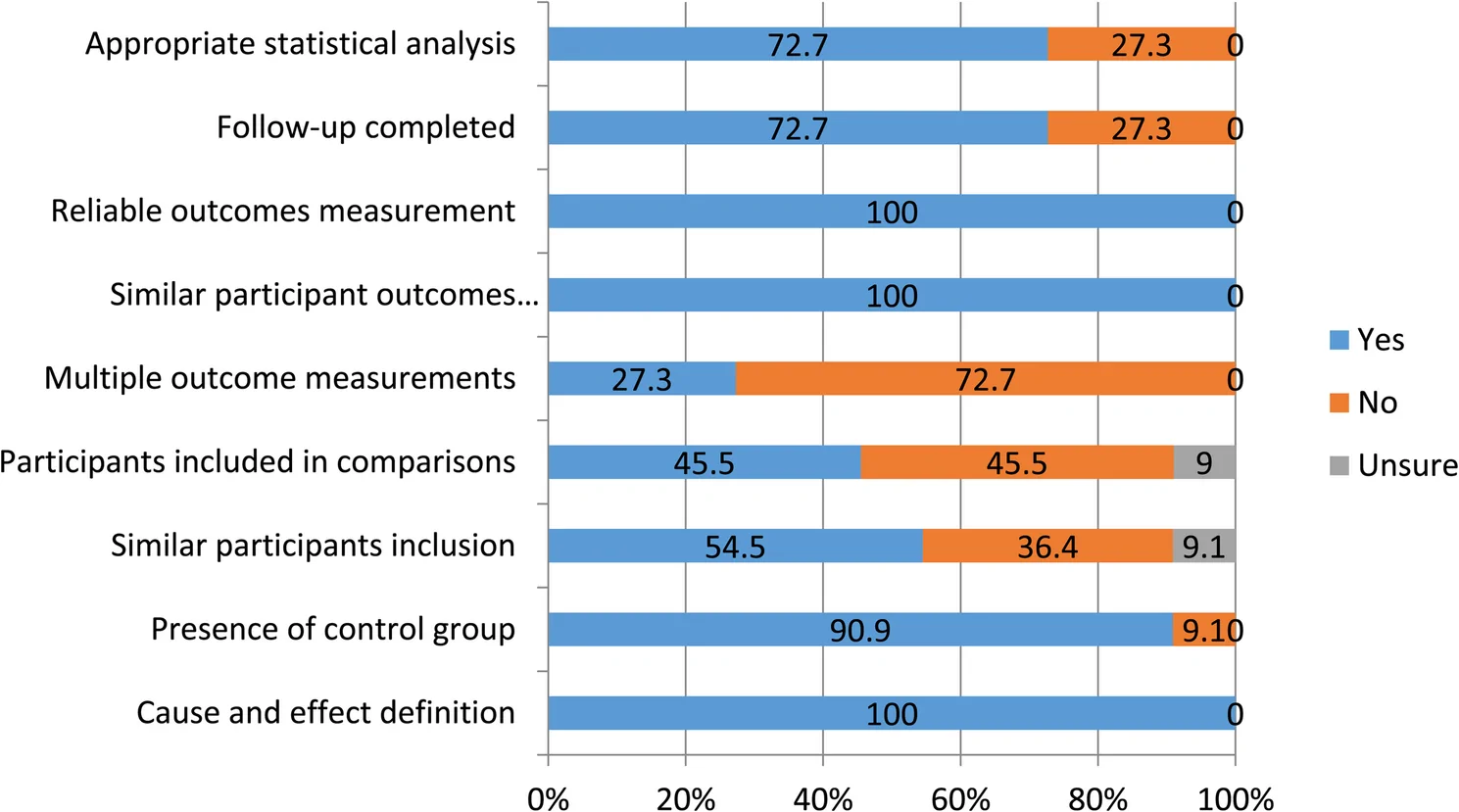
Quality assessment of included studies using the JBI checklist

The quality assessment/appraisal using JBI critical appraisals check list for quasi-experimental studies as presented in Fig. 2 showed that 100% of the reviewed studies had a clear definition of the cause and effect while majority (90.9%) had a control group. Based on the assessment, 54.5% of the included studies had intervention and comparator groups that were similar at baseline, while 45.5% reported that participants in the comparison groups received treatment similar to the intervention group, apart from the intervention being evaluated. All studies (100%) had their outcomes measured in a valid and reliable as well as the outcomes of participants’ while follow up and appropriate statistical analysis was 72.7%. In general, six (54.5%) were assessed as high quality, five records (45.5%) as medium quality and none as low quality, indicative of reliable evidence and low risk of bias.

### Intervention components and implementation strategies of selected studies

#### Interventions settings and mode of delivery

The intervention components and implementation strategies of the selected studies are shown in Table 2. All included studies implemented their interventions within school settings. Of the included studies, eight were conducted in primary schools [19–23, 25, 28, 29], two in secondary schools [24, 26], and one in a tertiary education institution [27].

**Table 2:** Intervention components and implementation strategies of selected studies

Study	Intervention	Basic care (control)	Settings/ mode of delivery	Duration of Intervention	frequency/intensity of intervention	Adherence support	Intervention Provider
Naidoo et al. [19]	Nutrition and Physical activity (NAP) intervention		Primary School	6 months		Integration of NAP intervention into School curriculum	School Educators
Draper et al. [20]	Physical activity intervention (Healthnutz)		Primary School	4 months		Follow-up focus group	teachers
Kemp and Pienaar [21]	Physical activity and behaviour modification component ( healthy eating habits, self-perception, methods to increase physical activity)		Primary School	13 weeks	Three times per day	A week long holiday program by recreation specialists	
Monyeki et al. [22]	Physical activity intervention		Primary School	10 months	Two 30 min exercise session per week	Sessions were supervised	Biokineticist
Regaieg et al. [23]	Aerobic exercise and physical education training session	1 session of 60-min physical education per week	Primary School	16 weeks	four 60 min aerobic exercise session per week	Supervision and standardized verbal encouragements	Exercise instructor
Maatoug et al. [24]	Physical Activity and Nutrition Intervention.	Group education session on healthy eating, snacking and self esteem	Secondary School	1 year	Twice a week physical session	Counselling from medical doctor, Dietitian and pediatrician	
Müller et al. [25]	Multi-dimentional school based physical activity intervention (physical activity, health and hygiene education, nutrition education)		Primary School	10 weeks	two 40 min Physical Education lesson per week	Intervention components were taught in collaboration with life orientation teachers.	life orientation teachers and Dancer instructors
Grace et al. [26]	Controlled trial of physical activity and nutrition education intervention		Secondary School	10 weeks	20 exercise sessions two days per week	Counselling from a registered dietitian	Biokineticist
Wanghi et al. [27]	Randomized trial on the effectiveness of simple education session on lifestyle change (Snacking, physical activity and combined diet and physical activity)		University	6 months		Counselling	Parents
Nqweniso et al. [28]	Physical activity, Health and hygiene education, Nutrition education with supplements	Deworming medication	Primary School	10 weeks	two weekly 40 min physical education lesson	Providing children with RUSF once a day. Intervention was also integrated in school curriculum.	Teachers and external physical exercise specialists
Idamokoro et al. [29]	Fundamental movement skills program		Primary School	9 weeks	Twice a week for 30 min	Supervision and recording of participants attendance in intervention log	researcher and research assistants

Interventions were delivered by diverse personnel, including trained school teachers, biokineticists, exercise instructors, dance instructors, parents, and research staff. In a good number of the studies, trained school teachers [19, 20, 25, 28] delivered the interventions. External intervention experts such as biokineticists [22, 26] exercise instructors [23, 28] and dance instructors [25] delivered some of the physical activity interventions. Furthermore, the parents were co-opted in delivering the intervention [27] parents in delivering the intervention, while in some instances this was facilitated by the researcher and a research assistant [29].

#### Types of interventions

Multicomponent interventions were predominant, with combined nutrition and physical activity interventions reported in the majority (63.6%) of studies [19, 21, 25–29]. In addition, few of the studies incorporated psychological and psychosocial aspects of the intervention [21, 24], which focused on improving self-perception and self-esteem through behavioural and psychological support activities, or captured health and hygiene education programmes aimed at increasing awareness of hygiene practices and communicable disease prevention [25, 28].

Specifically, these nutrition-related interventions studies [19, 21, 24–28] integrated distinctly or as part of other interventions, comprised mainly of general nutrition education programmes targeted for groups [19, 21, 25, 26, 28]. These nutrition education programs included lessons on healthy eating habits, food groups, balanced diets, portion control, and reducing sugar and fat intake while increasing fruit and vegetable consumption. Beyond nutrition education, several studies also incorporated personalized dietary counselling, in which participants received consultations tailored at improving the consumption of healthy diets [24]. On the other hand, energy-dense food supplementation through disbursement of peanut butter as satiety snack [27] and Ready-to-Use Supplementary Food (RUSF) [28] were reported in two studies. Also, food environment-related structural and policy changes focusing on increasing the availability of healthy foods and drinks (fruits, water, juices, low-fat foods) and reducing availability of unhealthy items (fizzy drinks, sweets, and chips) in school tuck-shops, alongside school nutrition policy development remained part of the nutrition intervention [19].

Physical activity was included as a core intervention in all studies, either as a standalone intervention [20, 22, 29] or in combination with other lifestyle interventions [19, 21, 23–28]. Structured exercise programmes were implemented in six studies [19–27, 29] and comprised aerobic exercise, strength and flexibility training, and sports skills development. Dance- or music-based physical activity was incorporated in four studies [25, 26, 28, 29], including traditional dance, dance lessons, and movement-to-music sessions. Walking or jogging interventions were reported in two studies [26, 27].

#### Frequency, duration and intensity of the interventions

The duration of the interventions lasted from a minimum of 9 weeks to a maximum of 1 year. The frequency and intensity of intervention delivery varied across studies and were reported for physical activity, educational, and nutritional supplementation components. Exercise sessions were most commonly delivered twice weekly [22, 24–26, 28], although frequencies of three sessions per week [21] and four sessions per week [23] were also reported, and exercise sessions lasted between 30 and 60 min.

Educational interventions, which comprised nutrition, health, physical, and behavioural education, lasted between 40 min and 1 h. Nutrition education sessions were delivered either weekly or as a series of structured classroom lessons [21, 26, 28]. Physical education was delivered through 40-minute sessions [25], while health education was delivered once weekly [20]. Behavioural education was also implemented weekly [21].

For the nutritional supplementation component of the studies, peanut butter supplements were used. Nqweniso et al. [28] provided participants with a sachet of Ready-to-Use Supplementary Food (RUSF) once a day which contained 530 kcal per 100 g sachet while participants in Wanghi et al. study consumed 70 g of peanut butter 5 times per week as afternoon snack.

### Study (Weight-Related) outcomes

Information on the anthropometric outcomes of the interventions for school aged children is presented in Table 3. While majority of the interventions had a comparator receiving basic/standard care [20, 22–26, 28, 29], the absence of a comparator or control group was observed in one study [19].

**Table 3:** Anthropometric outcome of the intervention

		Intervention				Comparator				
Author	Feature/indicator	*N*	Baseline	Final	Mean Change	*N*	Baseline	Final	Mean Change	Outcome interpretation
Naidoo et al. [19]	BMI^a^	Boys = 81	19.15 ± 0.52	19.95 ± 0.63	0.8					**Neutral Outcome;** BMI status increased but within normal threshold
	BMI^a^	Girls = 104	19.94 ± 0.37	20.59 ± 0.38	0.65					**Neutral Outcome;** BMI status increased but within normal threshold
Draper et al. [20]	Weight	Boys and girls = 423	35.8 ± 8.6	37.2 ± 8.7	1.4	Boys and girls = 85	36.9 ± 8.4	37.1 ± 8.4	0.2	**Unfavorable Outcome**: weight increased in both groups with greater increase observed in the intervention group than the comparator group.
	Weight		62 ± 13.17	59.12 ± 12.79	− 2.88	Boys = 7 Girls = 11 Total = 18	64.11 ± 12.28	66.40 ± 12.65	2.29	**Favorable Outcome**: weight for intervention group declined substantially, compared to elevated weight in control group
	BMI^a^	Boys = 7	27.52 ± 4.05	25.52 ± 4.06	− 2.00		27.82 ± 4.49	28.42 ± 4.56	0.60	**Favorable Outcome;** BMI status for intervention group declined substantially, compared to elevated status in control group
Kemp and Pienaar [21]	Waist Circumference	Girls = 13	89.38 ± 11.64	82.13 ± 10.02	-7.25*		91.05 ± 15.13	94.03 ± 15.67	2.98	**Favorable Outcome;** WC status for intervention group declined substantially, compared to elevated status in control group
	Mid Upper Arm Circumference	Total = 20	28.86 ± 3.44	25.65 ± 3.34	-3.21**		27.82 ± 3.28	29.57 ± 3.33	1.75	**Favorable Outcome;** MUAC status for intervention group declined substantially, compared to elevated status in control group
	Fat %		44.16 ± 8.88	38.45 ± 8.38	-5.71*		51.18 ± 13.49	55.32 ± 14.37	4.14	**Favorable Outcome;** Mean body fat% for intervention group declined substantially, compared to elevated status reported in control group
	BMI^a^	Boys = 36 (12 Years old)	20.68	20.36	-0.32	Boys = 34 (12 Years old)	20.39	18.77	-1.62	**Neutral Outcome**: Although the mean BMI decreased in the intervention group, the reduction was slightly smaller than that observed in the control group
Monyeki et al. [22]	BMI^a^	Boys = 39 (13 Years old)	18.39	17.36	-1.03	Boys = 11 (13 Years old)	16.79	14.48	-2.31	**Neutral Outcome**: Although the mean BMI decreased in the intervention group, the reduction was slightly smaller than that observed in the control group
	Body fat %	Boys = 36 (12 Years old)	20.68	20.36	0.32	Boys = 36 (12 Years old)	20.39	18.77	1.62	**Neutral Outcome**: Although the mean BMI decreased in the intervention group, the reduction was slightly smaller than that observed in the control group
		Boys = 39 (13 Years old)	18.39	17.36	1.01	Boys = 39 (13 Years old)	16.79	14.48	2.31	**Neutral Outcome**: Although the mean BMI decreased in the intervention group, the reduction was slightly smaller than that observed in the control group
	Weight		58.5 ± 8.8	59.2 ± 9.1	0.7		53.2 ± 11.9	55.8 ± 11.9	2.6	**Unfavorable outcome**: Weight increased across groups.
	BMI^a^		25.4 ± 3.1	24.8 ± 3.2**	-0.6		24.2 ± 3.8	24.7 ± 3.9	0.5	**Favorable outcome**: Mean BMI of the intervention group moved from overweight to normal category, with no reduction observed in the comparator group
Regaieg et al. [23]	Waist Circumference	Boys and girls = 14	81.5 ± 6.4	79.8 ± 6.2**	− 1.7	Boys and girls = 14	77.9 ± 8.5	78.6 ± 8.5	0.7	**Favorable outcome**: WC status for intervention group declined substantially, compared to elevated status in control group Positive.
	Fat Mass		33.1 ± 6.5	28.8 ± 6.9**	− 4.3		29.7 ± 6.7	29.5 ± 8.4	-0.2	**Favorable outcome**: FM status for intervention group declined substantially, compared to decline in the control group.
	Fat Free Mass		38.7 ± 4.5	41.8 ± 5**	3.1		37 ± 7.4	38.7 ± 6.8	1.7	**Favorable outcome**: FFM status for intervention group increased substantially, compared to the decline in the control group positive.
Maatoug et al. [24]	BMI for age z-score	Boy = 138 Girl = 179	1.89 ± 0.57	1.76 *±* 0.63	-0.13**	Boy = 98 Girl = 170	1.89 *±* 0.63	1.71 *±* 0.72	− 0.18**	**Neutral Outcome**: Although the mean BMI decreased in the intervention group, the reduction was smaller than that observed in the control group.
Müller et al. [25]	BMI for age z-score	Baseline = 300 Endline = 264	-0.1 ± 1.2	-0.1 ± 1.3	0	Baseline = 446 Endline = 255	0 ± 1.2	0.2 ± 1.3	-0.2	**Neutral outcome**: No change was observed in the intervention group and the control group increased slightly
Grace et al. [26]	BMI for age Z Score	Girls = 15 Boys = 7	2.46 ± 0.79	2.24 ± 0.89	-0.22**	Girls = 8 Boys = 11	2.10 ± 0.82	2.03 ± 0.83	-0.07	**Favorable outcome**: BMI for age status for intervention group declined substantially, compared to decline in the control group
Wanghi et al. [27]	**Weigh**t Diet Walk Diet and walk	Diet = 18 Walk = 25 Diet and walk = 21	69.6 ± 12.0 71.6 ± 15.5 72.1 ± 12.6	68.4 ± 11.9 70.8 ± 14.9 70.2 ± 12.1	− 1.14 ± 0.99** − 0.88 ± 1.05* -1.86 ± 1.2**	20	71.1 ± 11.2	71.9 ± 10.4	0.8 ± 3.98	**Favorable outcome**: weight for intervention group declined substantially, compared to the increase observed in the control group.
	**BMI for age Z score** Diet Walk Diet and walk		2.14 ± 0.72 1.99 ± 0.60 2.11 ± 0.57	1.96 ± 0.62 1.90 ± 0.58 1.88 ± 0.50	-0.17 ± 1.8** -0.16 ± 1.8* -0.22 ± 0.19**		1.91 ± 0.51	1.93 ± 0.49	+ 0.02 ± 0.01	**Favorable outcome**: BMI for age for intervention group declined substantially, compared to the increase observed in the control group.
	**BMI for age Z score** E1^b^=Physical activities and Deworming	Boys = 42	0.2 ± 1.0	0.2 ± 1.1	0.0	57	0.2 ± 0.8	0.3 ± 0.8	+ 0.1	**Neutral outcome**: No change was observed in the intervention group and the control group increased slightly
	**BMI for age Z score** E1^b^=Physical activities and Deworming	Girls = 42	0.4 ± 1.2	0.5 ± 1.1	+ 0.1	56	0.4 ± 1.3	0.5 ± 1.3	+ 0.1*	**Neutral outcome**: slight increase was observed across groups while remaining within normal range
	**BMI for age Z score** E2 ^b^ = Physical activities and Health and hygiene education intervention	Boys = 50	0.3 ± 1.3	0.3 ± 1.4	0.0	Boys = 45	0.2 ± 1.3	0.2 ± 1.3	0.0	**Neutral outcome**: No change was observed both groups
	**BMI for age Z score** E2 ^b^ = Physical activities and Health and hygiene education intervention	Girls = 49	0.0 ± 1.1	0.3 *±* 0.9	+ 0.3*	girls = 52	0.0 *±* 1.2	0.1 *±* 1.2	+ 0.1*	**Neutral outcome**: slight increase was observed across groups while remaining within normal range
Nqweniso et al. [28]	**BMI for age Z score** E3 ^b^ = Health and hygiene education intervention, Nutrition intervention and Deworming medication	Boys = 49	0.1 *±* 1.2	0.4 *±* 1.1	+ 0.3*	boys = 80	–0.2 *±* 1.1	0.0 *±* 1.2	-+0.2*	**Unfavorable outcome**: BMI for age for intervention group increased while that of the control group increased
	**BMI for age Z score** E3 ^b^ = Health and hygiene education intervention, Nutrition intervention and Deworming medication	Girls = 43	0.4 *±* 1.3	0.7 *±* 1.2	+ 0.3*	Girls = 71	0.3 *±* 1.1	0.4 *±* 1.0	+ 0.1*	**Neutral outcome**: increase was observed across groups while remaining within normal range
	**BMI for age Z score** E4 ^b^ = Physical activities, Health and hygiene education intervention, Nutrition intervention and Deworming medication	Boys = 89	–0.4 ± 1.1	0.0 *±* 1.1	-+0.4*	Boys = 46	–0.8 *±* 1.0	–0.8 *±* 1.1	+ 0.0	**Favorable outcome**: BMI for age for the intervention group declined substantially while the control group remained the same
	**BMI for age Z score** **E4** ^b^ **=** Physical activities, Health and hygiene education intervention, Nutrition intervention and Deworming medication	Girls = 81	–0.5 *±* 1.2	-0.2 *±* 1.2	-+0.3*	Girls = 40	–0.7 *±* 1.2	–0.5 *±* 1.2	-+0.2*	**Favorable outcome**: BMI for age for intervention group declined, compared to the decline observed in the control group.
	weight		24.00	23.84	0.16		23.66	24.07	-0.41	**Favorable outcome**: weight for intervention group declined, compared to the increase observed in the control group.
	Triceps skinfold (TSKF) (mm)		8.89	8.32	-0.57		9.43	8.31	-1.12	**Neutral Outcome**: TSKF reduced more in the control group compared to the intervention group.
	Subscapular skinfold (SSKF) (mm)		5.59	5.65	0.06		5.61	5.66	0.05	**Neutral Outcome**: There was no substantial changes in the SSKF across the groups.
Idamokoro et al. [29]	Waist Circumference	57	53.34	53.07	-0.27*	36	52.21	53.50	0.84	**Favorable outcome**: WC for intervention group declined significantly, compared to the increase observed in the control group.
	Hip Circumference		63.07	64.85	1.78		61.60	66.16	4.56	**Unfavorable outcome**: HC increased across groups.
	BMI		16.17	15.57	-0.57*		15.79	15.72	-0.07	**Favourable outcome**: BMI for intervention group declined significantly, compared to the decline in the control group.
	Fat %		12.82	12.49	-0.4		13.59	12.15	− 1.44	**Neutral Outcome**: fat% reduced more in the control group compared to the intervention group.
	skinfold		14.48	13.98	-0.5		15.04	13.53	-1.51	**Neutral Outcome**: SKF reduced more in the control group compared to the intervention group.

E1 ^b^ = Intervention arm 1 comprising physical activity and deworming

E2 ^b^ = Intervention arm 2 comprising physical activity and health and hygiene education

E3 ^b^ = Intervention arm 3 comprising health and hygiene education, nutrition intervention, and deworming medication

E4 ^b^ = Intervention arm 4 comprising physical activity, health and hygiene education, nutrition intervention, and deworming medication

**Significant at *P* < 0.01; *Significant at *P* < 0.05

Outcomes among participants with normal baseline anthropometric status were largely characterized by the maintenance of normal weight or body composition following the intervention, with only minor changes in anthropometric measures while remaining within the normal range [19, 22, 24, 25, 28]. Among studies without comparator groups, Naidoo et al. [19] reported slight increases in BMI, with mean increases of 0.80 kg/m² in boys and 0.65 kg/m² in girls, although participants remained within the normal BMI category.

Among studies with intervention and comparator groups, Draper et al. [20] reported a greater increase in body weight in the intervention group (**+** 1.40 kg) than in the comparator group (**+** 0.20 kg). Conversely, Monyeki et al. [22] and Maatoug et al. [24] reported reductions in BMI or BMI-for-age z-score in both groups, although the reductions were generally greater in the comparator groups. Across these studies, reductions in the intervention groups ranged from **−** 0.13 to − 1.03, compared with **−** 0.18 to − 2.31 in the comparator groups. Muller et al. [25] reported no change in BMI z-score in the intervention group (mean difference = 0.00) but a slight increase in the comparator group (**+** 0.20). In contrast, Nqweniso et al. [28] found greater reductions in BMI-for-age z-score among intervention participants (**−** 0.30 to − 0.40) than comparator participants (0.00 to − 0.20).

For participants who were predominantly overweight or obese at baseline, interventions more consistently produced clinically favourable reductions in adiposity. Among studies in which the intervention resulted in an improvement in weight status, reductions in BMI or BMI-for-age z-score ranged from **−** 0.22 to − 0.60 kg/m, with participants transitioning from the overweight/obese category to the normal weight range [23, 27] with comparator participants showing slight increases.

Other studies reported significant reductions in adiposity that, although clinically favourable, were insufficient to alter weight status. Across these studies, reductions in BMI or BMI z-score ranged from **−** 0.22 to − 2.00 kg/m^2^ in the intervention group than in the comparator group, with participants remaining in the overweight category at follow-up [21, 26].

### Meta-analysis of the effects of school-based interventions on BMI-for-age

**Fig. 3:**
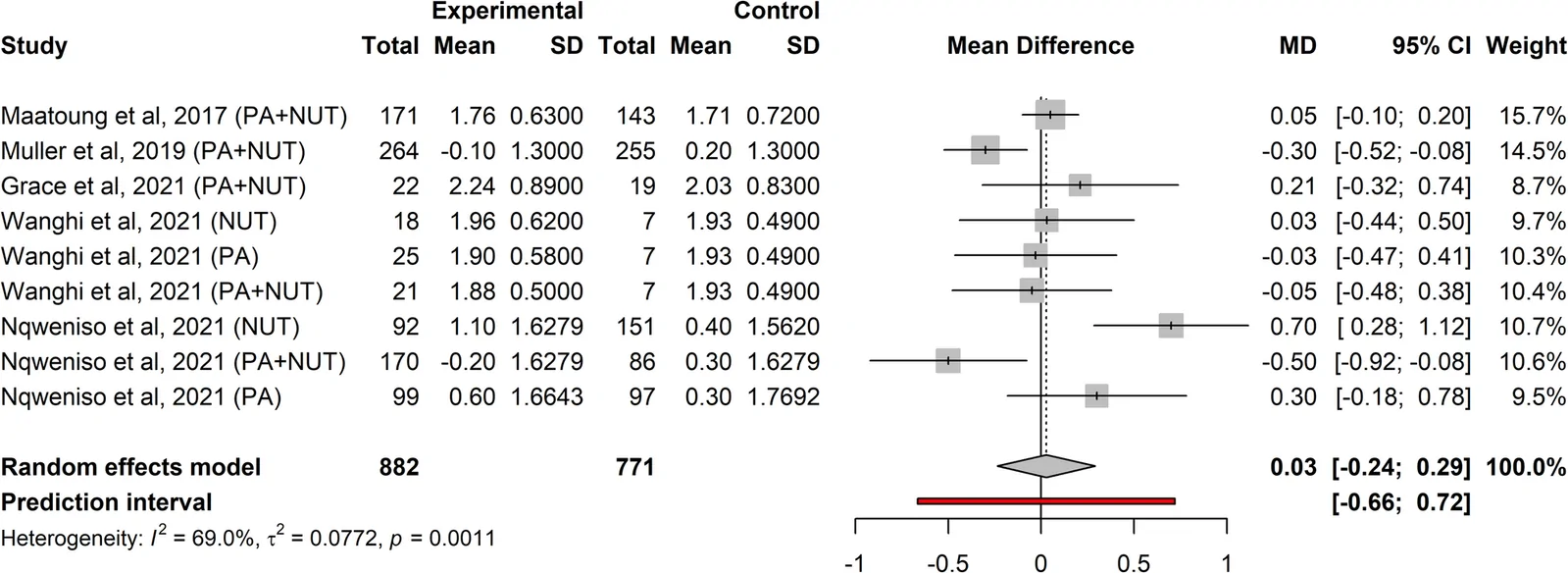
Forest plot of the pooled effects of school-based diet and physical activity interventions on BMI-for-age. PA: physical activity; NUT: nutrition; PA + NUT: combined physical activity and nutrition; MD: mean difference; CI: confidence interval. Negative MDs favour the intervention, indicating a lower BMI-for-age in the intervention group compared with the control group. Sex-specific data were combined where necessary, and shared control groups were adjusted to avoid double-counting. The pooled estimate was derived using a random-effects model.

Figure 3 illustrates the pooled effect of school-based diet and physical activity interventions on BMI-for-age. This quantitative synthesis comprised nine intervention comparisons from five studies [24–28], as two of the selected studies [27, 28] each had three intervention arms. Results showed that the various school-based lifestyle interventions were not associated with a statistically significant overall effect on BMI-for-age compared with control groups (MD = 0.03, 95% CI: −0.24 to 0.29; *p* = 0.813). In specifics, substantial between-study heterogeneity was observed (I² = 69.0%, Q = 25.78, *p* = 0.001). Multi-component interventions involving physical activity and nutrition combinations accounted for a significant decline in BMI-for-age in studies reported by Muller et al. [25] (MD = − 0.30, 95% CI: −0.52 to − 0.08) and Nqwensio et al. [28], (MD = − 0.50, 95% CI: −0.92 to − 0.08). In contrast, the nutrition intervention arm of the Nqweniso et al. [28] study reported a significant increase in BMI-for-age [28] (MD = 0.70, 95% CI: 0.28 to 1.12). A number of the remaining intervention comparisons were not statistically significant.

## Discussion

This systematic review and meta-analysis examined 11 school-based obesity intervention studies implemented and evaluated across several African countries, with the majority conducted in South Africa as reported by Adom [13]. This indicates limited evidence on the effectiveness of obesity interventions in other regions of Africa.

The majority of the studies included in the review employed multicomponent interventions comprising physical activity and nutrition education, with a few also incorporating behavioural support and supplementation. Physical activity was the core intervention across all studies and was primarily targeted at reducing obesity. Interventions combining physical activity and nutrition education reduced anthropometric measures among participants who were overweight or obese, while helping maintain anthropometric status among those with normal measurements. The greater effectiveness of the combined approach is consistent with the findings of Faiz et al. [30] and Salem et al. [31]. It may be attributed to its complementary effects on energy balance, whereby physical activity increases energy expenditure while nutrition education promotes healthier dietary choices, portion control and reduced energy intake. Furthermore, multicomponent interventions that incorporated behavioural support, active supervision, and follow-up were successful. Angawi and Gaissi [34] also reported that interventions that considered both physical activity (PA) and diet, along with close monitoring, were highly effective in reducing obesity.

Interventions lasting at least 9 weeks and involving at least two physical activity sessions per week were associated with significant improvements in anthropometric outcomes, a finding consistent with evidence that repeated aerobic exercise over several weeks can reduce body weight, waist circumference, body-fat percentage, visceral adipose tissue and subcutaneous adipose tissue, with greater exercise exposure generally producing greater reductions [32]. However, the predominantly neutral anthropometric effects observed among interventions lasting 6 months or longer may reflect an attenuation of anthropometric change over time rather than a lack of intervention effectiveness. Weight reduction does not necessarily continue at the same rate throughout prolonged interventions, as weight loss often slows and approaches a plateau after the initial months of lifestyle treatment [33]. At the same time, physiological feedback mechanisms increasingly oppose continued weight loss as body weight declines [34]. In addition, compensatory responses to exercise, including adjustments in energy intake and expenditure, may become more pronounced with longer intervention durations thereby reducing further changes in body weight and adiposity despite continued physical activity [35]. Baseline anthropometric status may also contribute to this pattern, as exercise-induced reductions in adiposity tend to be more pronounced among individuals with overweight or obesity than among those with healthier baseline profiles [36]. Consequently, where participants were already within normal anthropometric ranges, stability in body weight, BMI or related measures during longer interventions may reasonably reflect maintenance of a favourable anthropometric status rather than intervention failure. Also, this suggests that the effectiveness of an intervention may be more closely related to its components, intensity, and delivery than to duration alone.

Although the low risk of bias benchmark is a consequence of heterogeneity of study designs of included studies, the eventual relatively modest risk-of-bias ratings reported may be due to the predominance of quasi-experimental designs in this review. Other experimental designs particular RCTs have been shown to offer stronger protection against selection and confounding bias than quasi-experimental designs which could increases the risk of methodological bias [15, 37]. Furthermore, the review also revealed disparities in the measurement and interpretation of anthropometric outcomes, with some studies, particularly older studies, reporting BMI rather than age- and sex-adjusted BMI-for-age. This may reflect the gradual adoption of standardised growth references in research involving children and adolescents, as previously observed by Iheme [38]. Such measurement differences may have contributed to variations in reported intervention effects, since using unadjusted BMI can either overestimate or underestimate changes in weight status during periods of growth. This highlights the need for greater adoption of standardised age-appropriate measures, such as BMI-for-age, to improve the comparability and interpretation of school-based obesity intervention outcomes.

## Conclusion

This systematic review and meta-analysis indicates that school-based obesity interventions incorporating physical activity can contribute to improvements in anthropometric outcomes among children and adolescents in Africa, particularly when physical activity is combined with nutrition education and other supportive strategies. Across the 11 included studies, multicomponent interventions were generally more favourable for reducing anthropometric measures among participants who were overweight or obese, while stability in anthropometric measures among participants with normal baseline values may indicate maintenance of a healthy weight status rather than absence of intervention benefit. Overall, the findings suggest that intervention effectiveness may depend more on the combination of intervention components, frequency and intensity of physical activity, participant baseline weight status, behavioural support, supervision and quality of programme delivery than on intervention duration alone. Although the meta-analysis showed no significant overall effect on BMI-for-age, combined nutrition and physical activity interventions demonstrated reductions in BMI-for-age in some comparisons.

School-based multicomponent interventions therefore represent a promising approach for addressing overweight and obesity among African children and adolescents. However, stronger and more geographically representative evidence using standardized measure of weight status is required before firm conclusions can be drawn for the continent as a whole. In addition, there is need to shift attention of outcomes in school-based intervention studies targeted for populations with predominantly normal weight predominant population from weight-related outcomes to the formation of positive healthy lifestyles. In addition, school-based intervention studies conducted among predominantly normal-weight populations should shift their focus from weight-related outcomes towards the development and maintenance of healthy lifestyle behaviours.

### Study Limitations

The findings of this review should be interpreted in light of several limitations. First, most were conducted in a small number of African countries, particularly South Africa. This geographical concentration limits the generalisability of the findings to other African. Second, there was substantial variation in participants’ baseline anthropometric status. Differences in BMI and weight status could influence the magnitude of intervention effects because participants who were overweight or obese had greater potential for reductions in body weight and adiposity than those who were already within normal anthropometric ranges. In studies involving children and adolescents, differences in the use and reporting of age- and sex-adjusted measures, particularly BMI-for-age, may further affect comparability between studies.

Finally, not all studies included in the systematic review could be incorporated into the meta-analysis because some did not meet the statistical or outcome-reporting requirements for quantitative synthesis. Reasons included the absence of an appropriate comparator group, failure to report BMI-for-age or sufficiently comparable anthropometric outcomes. This reduced the number of studies contributing to the pooled analyses and consequently limited the statistical power and precision of the meta-analytic estimates. The relatively small evidence base further limits the ability to adequately investigate subgroup effects and publication bias. Future studies should therefore adopt standardised age-appropriate anthropometric measures and expand research to a wider range of African countries.

## Supplementary Information


Supplementary Material 1.


## Data Availability

Availability of data and material: Data used in this study are available to the authors and can be shared upon reasonable request.

## Abbreviations

WHO: World health Organisation
LMICs: Low- and medium-income countries
DALYs: Disability-adjusted life years
PRISMA: Preferred Reporting Items for Systematic Reviews and Meta-Analyses
AJOL: African Journals Online
PICOS: Population, Intervention, Comparison, Outcome and Study Design
JBI: Joanna Briggs Institute
RCT: Randomized control trial
BMI: Body mass index
WC: Waist circumference
MUAC: Mid-Upper arm circumference
HC: Hip Circumference
FM: Fat mass
FFM: Fat free mass
TSKF: Triceps skinfold
SSKF: Subscapular skinfold
SKF: Skin fold
NAP: Nutrition and Physical Activity
PAR-Q: Physical Activity Readiness Questionnaire
GPAQ: Global Physical Activity Questionnaire
MAP: Mean arterial pressure
PP: Partial pressure
HDL: High density lipoprotein
LDL: Low density lipoprotein
TG: Triglyceride
TC: Total cholesterol
MVPA: Moderate-to-vigorous physical activity
PA: Physical activity
SAC: School aged children
FIA: Feel- minus-idea
TFT: Task-oriented functional training
BIA: Bioelectrical impedance analysis
HHE: Health and hygiene education
N: Nutrition intervention
DE: Deworming medication
