# Supplementary material for: School-based diet and physical activity interventions and weight-related outcomes among children and adolescents in Africa: a systematic review and meta-analysis
Source: BMC Public Health. 2026 Aug 29;26:2484. doi: 10.1186/s12889-026-29255-3 (PMC13526300; doi:10.1186/s12889-026-29255-3)
Supplement: Supplementary file 1 [file 12889_2026_29255_MOESM1_ESM.docx]

**Supplementary table 1- search strategy**

Pubmed search strategy for intervemtion

| **Search Terms** | **Hits** |  |
| --- | --- | --- |
| children |  |  |
| ("Child"[Mesh]) OR "Adolescent"[Mesh] OR "School-aged children" | 370.127 |  |
| school |  |  |
| **("Schools"[Mesh]) OR "School Health Services"[Mesh] OR "school-based" or "School intervention"** | 17,106 |  |
| Physical activity |  |  |
| **"Exercise"[Mesh] OR Motor Activity OR Physical Fitness OR Sports OR physical activity** | 153,374 |  |
| Nutrition intervention |  |  |
| **(("Nutritional Status"[Mesh]) OR "Diet"[Mesh]) OR "Diet Therapy"[Mesh] OR Nutrition education** | 63,560 |  |
| **Anthropometric outcome** |  |  |
| **((("Body Weight"[Mesh]) AND "Body Mass Index"[Mesh]) OR "Obesity"[Mesh]) OR "Overweight"[Mesh] OR weight come OR BMI** | 66,313 |  |
| **Africa** |  |  |
| **((((((("Africa"[Mesh]) OR "Africa South of the Sahara"[Mesh]) OR "South Africa"[Mesh]) OR "Africa, Western"[Mesh]) OR "Africa, Southern"[Mesh]) OR "Africa, Northern"[Mesh]) OR "Africa, Eastern"[Mesh]) OR "Africa, Central"[Mesh]** | 34,379 |  |
| **(((((("Child"[Mesh]) OR "Adolescent"[Mesh] OR "School-aged children") AND (("Schools"[Mesh]) OR "School Health Services"[Mesh] OR "school-based" or "School intervention")) AND ("Exercise"[Mesh] OR Motor Activity OR Physical Fitness OR Sports OR physical activity)) AND ((("Nutritional Status"[Mesh]) OR "Diet"[Mesh]) OR "Diet Therapy"[Mesh] OR Nutrition education)) AND (((("Body Weight"[Mesh]) AND "Body Mass Index"[Mesh]) OR "Obesity"[Mesh]) OR "Overweight"[Mesh] OR weight come OR BMI) ) AND (((((((("Africa"[Mesh]) OR "Africa South of the Sahara"[Mesh]) OR "South Africa"[Mesh]) OR "Africa, Western"[Mesh]) OR "Africa, Southern"[Mesh]) OR "Africa, Northern"[Mesh]) OR "Africa, Eastern"[Mesh]) OR "Africa, Central"[Mesh])** | 10 |  |
|  |  |  |

Search string for Children AND school-based AND physical activity AND nutrition intervention AND anthropometric outcome

(("Child"[MeSH Terms] OR "Adolescent"[MeSH Terms] OR "School-aged children"[All Fields]) AND ("Schools"[MeSH Terms] OR "School Health Services"[MeSH Terms] OR "school-based"[All Fields] OR "School intervention"[All Fields]) AND ("Exercise"[MeSH Terms] OR ("motor activity"[MeSH Terms] OR ("motor"[All Fields] AND "activity"[All Fields]) OR "motor activity"[All Fields]) OR ("physical fitness"[MeSH Terms] OR ("physical"[All Fields] AND "fitness"[All Fields]) OR "physical fitness"[All Fields]) OR ("sport s"[All Fields] OR "sports"[MeSH Terms] OR "sports"[All Fields] OR "sport"[All Fields] OR "sporting"[All Fields]) OR ("Exercise"[MeSH Terms] OR "Exercise"[All Fields] OR ("physical"[All Fields] AND "activity"[All Fields]) OR "physical activity"[All Fields])) AND ("Nutritional Status"[MeSH Terms] OR "Diet"[MeSH Terms] OR "Diet Therapy"[MeSH Terms] OR (("nutrition s"[All Fields] OR "Nutritional Status"[MeSH Terms] OR ("nutritional"[All Fields] AND "status"[All Fields]) OR "Nutritional Status"[All Fields] OR "nutrition"[All Fields] OR "nutritional sciences"[MeSH Terms] OR ("nutritional"[All Fields] AND "sciences"[All Fields]) OR "nutritional sciences"[All Fields] OR "nutritional"[All Fields] OR "nutritionals"[All Fields] OR "nutritions"[All Fields] OR "nutritive"[All Fields]) AND ("educability"[All Fields] OR "educable"[All Fields] OR "educates"[All Fields] OR "education"[MeSH Subheading] OR "education"[All Fields] OR "educational status"[MeSH Terms] OR ("educational"[All Fields] AND "status"[All Fields]) OR "educational status"[All Fields] OR "education"[MeSH Terms] OR "education s"[All Fields] OR "educational"[All Fields] OR "educative"[All Fields] OR "educator"[All Fields] OR "educator s"[All Fields] OR "educators"[All Fields] OR "teaching"[MeSH Terms] OR "teaching"[All Fields] OR "educate"[All Fields] OR "educated"[All Fields] OR "educating"[All Fields] OR "educations"[All Fields]))) AND (("Body Weight"[MeSH Terms] AND "Body Mass Index"[MeSH Terms]) OR "Obesity"[MeSH Terms] OR "Overweight"[MeSH Terms] OR (("weight s"[All Fields] OR "weighted"[All Fields] OR "weighting"[All Fields] OR "weightings"[All Fields] OR "weights and measures"[MeSH Terms] OR ("weights"[All Fields] AND "measures"[All Fields]) OR "weights and measures"[All Fields] OR "weight"[All Fields] OR "Body Weight"[MeSH Terms] OR ("body"[All Fields] AND "weight"[All Fields]) OR "Body Weight"[All Fields] OR "weights"[All Fields]) AND "come"[All Fields]) OR "BMI"[All Fields]) AND ("Africa"[MeSH Terms] OR "Africa South of the Sahara"[MeSH Terms] OR "South Africa"[MeSH Terms] OR "africa, western"[MeSH Terms] OR "africa, southern"[MeSH Terms] OR "africa, northern"[MeSH Terms] OR "africa, eastern"[MeSH Terms] OR "africa, central"[MeSH Terms])) AND ((fha[Filter]) AND (clinicalstudy[Filter] OR comparativestudy[Filter] OR evaluationstudy[Filter] OR multicenterstudy[Filter] OR observationalstudy[Filter] OR randomizedcontrolledtrial[Filter] OR validationstudy[Filter]) AND (2000:2026[pdat]))

**Supplementary table 2**

**Summary of Study Characteristics**

| **Characteristics** | **N (%)** | **References** |
| --- | --- | --- |
| **Publication year** |  |  |
| 2002 – 2012 | 4 (36.4) | [16–19] |
| 2013 – 2022 | 7 (63.6) | [21,22,26,28,29,30,33] |
| **Country** |  |  |
| DR Congo | 1 (9.1) | [29] |
| South Africa | 8 (72.7) | [16–19,26,28,30,33] |
| Tunisia | 2 (18.2) | [21,22] |
| **Number of participants** |  |  |
| Less than 100 | 5 (45.5) | [18,21,28,29,33] |
| 101 – 1000 | 5 (45.5) | [16,17,19,26,30] |
| 1001 and above | 1 (9.1) | [21] |
| **Sex** |  |  |
| Studies with males and females | 10 (90.9) | [16–18,21,22,26,28–30,33] |
| Males – only study | 1 (9.1) | [19] |
| **Study designs/techniques** |  |  |
| Quasi-experimental | 8 (72.7) | [16–19,21,22,28,33] |
| Randomized Control Trial | 1 (9.1) | [29] |
| Cluster RCT | 2 (18.2) | [26,30] |
